# *Opuntia stricta* (Haw.) Fruit Pulp and Seeds as Source of Bioactive Phytochemicals with Promising Functional Properties

**DOI:** 10.3390/molecules30071580

**Published:** 2025-04-01

**Authors:** Roberta Pino, Natale Badalamenti, Stefano Dall’Acqua, Rosa Tundis, Maurizio Bruno, Francesco Sottile, Stefania Sut, Monica Rosa Loizzo

**Affiliations:** 1 Department of Pharmacy, Health and Nutritional Sciences, University of Calabria, 87036 Rende, Italy; roberta.pino@unical.it (R.P.); rosa.tundis@unical.it (R.T.); monica_rosa.loizzo@unical.it (M.R.L.); 2 Department of Biological, Chemical and Pharmaceutical Sciences and Technologies (STEBICEF), University of Palermo, Viale delle Scienze, 90128 Palermo, Italy; maurizio.bruno@unipa.it; 3 NBFC—National Biodiversity Future Center, 90133 Palermo, Italy; 4 Department of Pharmaceutical and Pharmacological Sciences, University of Padova, via F. Marzolo 5, 35131 Padova, Italy; stefania.sut@unipd.it; 5 Centro Interdipartimentale di Ricerca “Riutilizzo Bio-Based Degli Scarti da Matrici Agroalimentari” (RIVIVE), University of Palermo, Viale delle Scienze, 90128 Palermo, Italy; francesco.sottile@unipa.it; 6 Department of Architecture, University of Palermo, Viale delle Scienze, 90128 Palermo, Italy

**Keywords:** *Opuntia stricta*, betalains, antioxidants, obesity, functional food, nutraceutical products

## Abstract

In recent years, *Opuntia stricta* (Cactaceae family) has garnered considerable attention due to its promising nutritional and medicinal properties. This study aims to investigate the chemical composition and bioactivity of Sicilian *Opuntia stricta* fruit pulp and seeds. Liquid chromatography–mass spectrometry analysis revealed the presence of betalain derivatives, especially isobetanin and betanin, as the main pigments in the freeze-dried pulp and its hydroalcoholic extract. Other constituents, namely, piscidic acid, isorhamnetin-3-*O*-glucoside, and isorhamnetin-3-*O*-rutinoside, were identified. Linoleic acid (41.95%) was the main abundant fatty acid followed by palmitic acid (19.32%) in the seed’s fixed oil as analyzed by gas chromatography–mass spectrometry. The antioxidant activity was assessed using a multi-target approach using 2,2-azinobis (3-ethylbenzothiazoline-6-sulfonic acid) (ABTS), 2,2-diphenyl-1-picrylhydrazyl (DPPH), ferric reducing antioxidant power assay (FRAP), and *β*-carotene bleaching tests. The ABTS test showed greater sensitivity to the action of the samples with significant half-maximal inhibitory concentrations (IC_50_) of 13.24 and 14.82 mg/mL for the hydroalcoholic extract and the freeze-dried fruit pulp, respectively. *Opuntia stricta*’s extracts were also assessed for the carbohydrate-hydrolyzing enzyme and lipase inhibitory effect. The freeze-dried fruit pulp exhibited the highest effect against lipase (IC_50_ of 33.54 μg/mL). Collectively, our results contribute to the characterization of this traditionally consumed Sicilian edible plant and suggest its use as a source of bioactive compounds useful for the prevention of obesity linked to hyperglycemia.

## 1. Introduction

Recently, plants from the Cactaceae family, particularly those of the *Opuntia* genus, have gained increasing attention due to their therapeutic and nutritional potential.

*Opuntia* species are stress tolerant (e.g., salinity, drought), succulent CAM plants with a highly efficient water use. For this reason, plants from this genus are widely distributed in Mediterranean and semiarid areas [1].

Among these, *Opuntia stricta*, also known as “prickly pear”, stands out for its resistance to harsh climatic conditions and its variety of bioactive compounds. Growing in arid environments, *Opuntia stricta* has developed a rich chemical biodiversity, which provides it with numerous beneficial effects, including antioxidant, anti-inflammatory, and antimicrobial properties. This plant is known for its various agricultural and therapeutic applications, thanks to the unique chemical composition of its fruits, flowers, and stems [2]. In southern regions, many species have been reported to survive and produce fruit in arid environments where water availability is a limiting factor. This is particularly significant today, given the ongoing climate crisis that makes water one of the most at-risk non-renewable resources. For this reason, species belonging to the *Opuntia* genus are considered strategic, especially when they are relevant as food; this is the case for *Opuntia ficus-indica*, which has garnered widespread interest globally for the consumption of its fruits and other plant derivatives. However, it is important not to underestimate the role that many of these Cactaceae species can play ecologically in mitigating the effects of the climate crisis and contributing to the potential achievement of sustainable development goals, such as the strengthening of biodiversity conservation and the development of sustainability models. For example, *Opuntia stricta* (Haw.) Haw. [syn. *Opuntia dillenii* (Ker-Gawl) Haw.] has garnered significant attention as a species with extensive adaptive plasticity. It has been extensively studied as a model species in invasive ecological processes to demonstrate the effects of land-use changes on its spread, particularly in relation to grazing management and the presence of wildlife [3]. At the same time, *Opuntia stricta* produces a significant number of fruits, which, although not particularly interesting from a dietary perspective, contain important bioactive substances that may support the development of extraction models for nutraceutical purposes [4].

Moreover, the root system’s ability to mitigate hydrogeological risk, combined with drought resistance, gives these species enormous ecological potential. *Opuntia* spp. have also been reported to contribute to carbon sequestration from the atmosphere through deposition in the soil, a strategically important aspect in today’s actions to mitigate atmospheric gases that alter the climate, by reducing carbon dioxide concentrations [5].

Several scientific studies have explored the intricate relationship between obesity and type 2 diabetes [6]. Obesity is one of the most important and significant risk factors in the development and progression of type 2 diabetes mellitus (T2DM) regardless of age group. As of 2021, approximately 1.1 million individuals aged 65 to 74 and about 1.4 million aged 75 and over were affected by diabetes mellitus in Italy. Notably, around 14.1% of obese men in Italy had been diagnosed with diabetes as of 2021, highlighting the strong association between obesity and type 2 diabetes in the Italian population [7].

This condition has reached pandemic proportions, making the treatment of obesity crucial in the prevention and management of type 2 diabetes mellitus worldwide. Numerous clinical studies have shown that weight loss in association with the use of food supplements of natural origin can improve blood glucose levels and insulin action, especially in patients without full-blown T2DM [8].

In the pathogenesis and progression of T2DM, insulin plays a role in reducing oxidative stress. In fact, insulin resistance, chronic hyperglycemia, and mitochondrial dysfunction can facilitate the production of reactive oxygen species (ROS) which cause cellular damage and dysfunction, further exacerbating oxidative stress. Among the cells most affected are the endothelial ones, hence the development of diabetic vascular complications such as retinopathy, nephropathy, and cardiovascular diseases. ROS are also responsible for diabetic nephropathy, neuropathy, and retinopathy. Strategies to mitigate oxidative stress in diabetes include the administration of antioxidants which contribute to the control of the pathology [9].

Recent studies have highlighted the importance of *Opuntia* species as sources of bioactive compounds, with potential applications in the nutraceutical and pharmaceutical fields.

Specifically, a study by Loukili et al. [10] analyzed the chemical composition and physicochemical properties of extracts from *Opuntia dillenii* cultivated in Morocco. The results revealed the presence of important bioactive compounds, including flavonoids, phenolic acids, and anthocyanins, which provide the plant with significant antioxidant, anti-inflammatory, and antidiabetic properties. These findings suggest that *Opuntia stricta* could be a valuable resource for the development of new natural remedies and dietary supplements.

*Opuntia stricta* possesses an interesting chemical composition, characterized by bioactive compounds that justify its antioxidant potential. Moreover, the presence of antimicrobial compounds also suggests that the extracts could be used in cosmetic applications or as natural additives in food products to extend their shelf life [11].

*Opuntia stricta* has also attracted scientific attention for its antidiabetic properties, due to the presence of bioactive compounds. The oil extracted from its seeds is being studied for its therapeutic potential, including in the regulation of blood sugar levels and in its protective action against oxidative stress, a key factor in the development and progression of diabetes. In vivo studies show effects on the entire body, such as reduced blood glucose levels and insulin resistance, while in vitro and ex vivo studies explore its action on specific biological targets, such as metabolic enzymes and the antioxidant system [12].

The biodiversity of Sicily’s flora is rich and unique, reflecting the island’s diverse landscapes and Mediterranean climate. Sicily is home to more than 2000 plant species, many of which are endemic, meaning they are found nowhere else in the world. This includes a variety of flowering plants, shrubs, and trees that thrive in the island’s coastal areas, mountains, and inland regions. The island’s flora is influenced by its varied climate zones, from temperate coastal areas to the more arid inland regions, as well as by its complex geological history. This diversity of plant life makes Sicily a key region for plant conservation and ecological research [13].

The aim of this paper is the chemical characterization of the fruit pulp and seed fixed oil from *Opuntia stricta* collected in Sicily with the aim of highlighting its distinctive character in terms of chemical composition, considering the biodiversity of the flora of the Sicilian Territory and identifying its nutraceutical potential.

## 2. Results and Discussion

### 2.1. Total Phenol and Flavonoid Content

Phenolic compounds are plant secondary metabolites known in the literature for their biological effects, including antioxidant, anti-inflammatory, antimicrobial, hypoglycemic cardioprotective, neuroprotective, etc. [14,15,16]. *Opuntia* species are rich in phenolic compounds. Among them, more than 40 compounds are found in both the pulp and cladodes and more than 20 in the seeds [17,18].

Table 1 reports the total phenols (TPC) and flavonoids (TFC) in *Opuntia stricta* fruit pulp and its hydroalcoholic extract. Generally, the dried fruit pulp samples exhibited a higher TPC and TFC with values of 546.09 mg of the chlorogenic acid equivalent (CAE)/g fresh weight (FW), and 276.02 mg of the quercetin equivalent (QE)/g FW, respectively.

Previously, Shirazinia et al. [19] determined the TPC in Iranian *Opuntia dillenii* fruit’s hydroalcoholic extract (EtOH:H_2_O 50:50 *v*/*v*) and found a value of 65 mg GAE/g dried extract.

More recently, Marhri et al. [20] compared the TPC and TFC of the fruit peel of three Moroccan *Opuntia* species: *dillenii, ficus-indica*, and *robusta*. *Opuntia robusta* exhibited the highest TPC and TFC with values of 5583.19 mg GAE/100 g and 21,041.03 μg/g, respectively. A moderate TPC was detected in *Opuntia dillenii* (5513.55 mg GAE/100 g). Several Portuguese *Opuntia* species fruits were investigated for their TPC [21]. Generally, *Opuntia dillenii* showed the highest TPC with a mean TPC value of 3596.5 mg GAE/kg FW, followed by *Opuntia elata.* The Tunisian prickly pears *Opuntia ficus-indica* (spiny and thornless forms) and *Opuntia stricta* have been studied for their TPC and TFC. Results evidenced that the TPC is higher in the *Opuntia* peel than in the pulp; moreover, among investigated species, it is higher in *Opuntia ficus-indica* than in *Opuntia stricta*. These results agree with those found by Díaz Medina et al. [22] and Moussa et al. [23]. Values of 1.6 g/100 g and 197 mg/100 g were recorded for TPC and TFC in *Opuntia stricta* whole fruit from Kenya [24].

### 2.2. Chemical Profile of Opuntia Stricta Freeze-Dried Fruit Pulp (OSF) and Its Hydroalcoholic Extract (OSC)

Red pigments are present in both the extracts, and quantitative analysis of the total pigment was performed by UV–Vis. The analysis revealed the presence of 10.4 and 6.0% of betanin in the freeze-dried fruit pulp and its hydroalcoholic extract, respectively. The higher pigment content of the hydroalcoholic extract can be explained by the precipitation of mucilage due to the hydroalcoholic extraction procedure and its removal by filtration.

**Figure 1:**
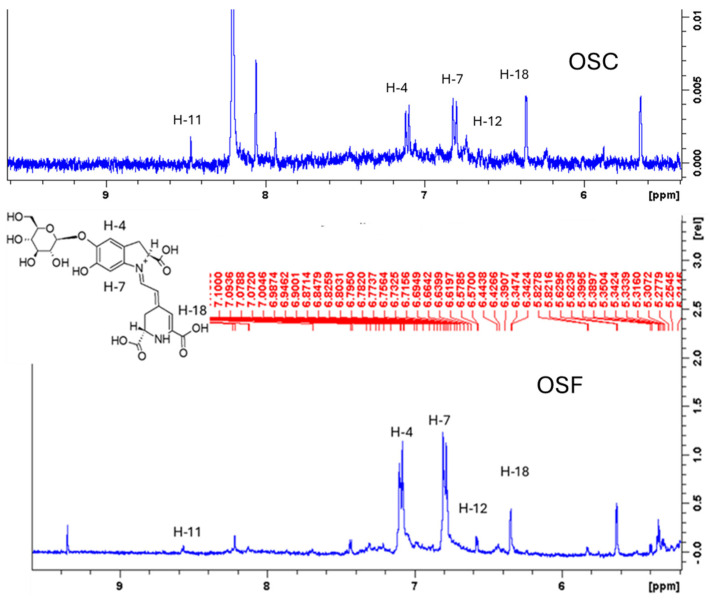
Enlarged region between 5 and 9 ppm of ^1^H NMR of the freeze-dried fruit pulp and its hydroalcoholic extract.

The *Opuntia stricta* fruit pulp extract samples were preliminarily analyzed by ^1^H-NMR dissolving in water. The main compounds detected were glucose (alpha and beta forms) while small peaks were observed in the aromatic region ascribable to betanin derivatives at *δ* 7.11 and 7.09 corresponding to the CH-4 and CH-7, respectively, in agreement with previously reported data [25]. Betanines were detected in both investigated samples. The ^1^H-NMR of the freeze-dried fruit pulp is reported in Figure 1; the signals related to the H-4 H-7, H-18, H-11, and H-12 are highlighted. Thus, the ^1^H-NMR results allowed for the detection of betalain derivatives in the *Opuntia stricta* samples.

**Figure 2:**
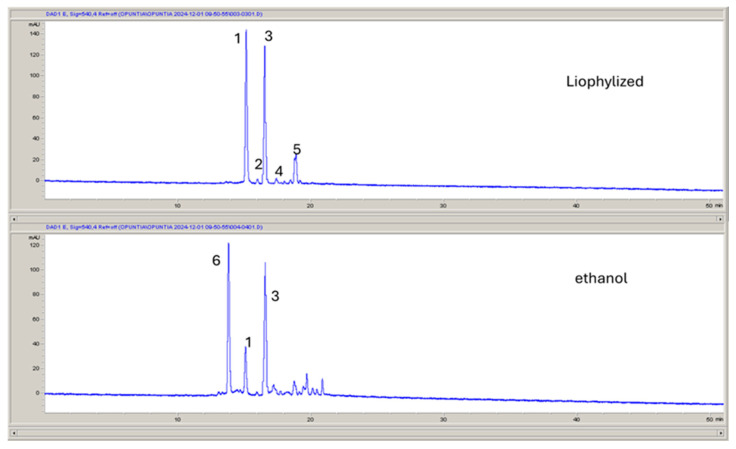
Chromatograms of freeze-dried fruit pulp (OSF) and its hydroalcoholic extract (OSC) at 540 nm (1: betanin; 2: decarboxy betanin isomer 1; 3: isobetanin; 4: decarboxy betanin isomer 2; 5: decarboxy betanin isomer 3; 6: decarboxy betanin isomer 4).

The samples were then subjected to liquid chromatography–mass spectrometry analysis to increase the information on the pigments. The extracts presented different chromatograms, which are reported in Figure 2. Six main peaks present the UV maximum at 540 nm and show the typical spectrum of betalain derivatives.

Identification was obtained combining data from multiple stage mass spectrometry (MS^n^). Two peaks (1 and 3) present molecular ions [M + H]^+^ at *m*/*z* 551, and three peaks (2, 4, and 5) present [M + H]^+^ at *m*/*z* 507, shown in Figure S1 (Supplementary Materials). The fragmentation of the species at *m*/*z* 551 leads to an intense peak at *m*/*z* 389 in MS^2^ and further fragments at *m*/*z* 343, 297, and 258 in MS^3^. Data are in agreement with a previously published paper on the ESI-MS identification of betanins in *Opuntia* species [26].

A further intense peak with the same UV maximum is observed at 13.7 min in the hydroalcoholic extract. This peak presents similar UV data as the other betalains but a different *m*/*z* value, namely, 318, and an isotopic pattern that shows the ion to be double charged. This derivative is formed during the concentration procedures due to the temperature increase and the presence of ethanol.

**Figure 3:**
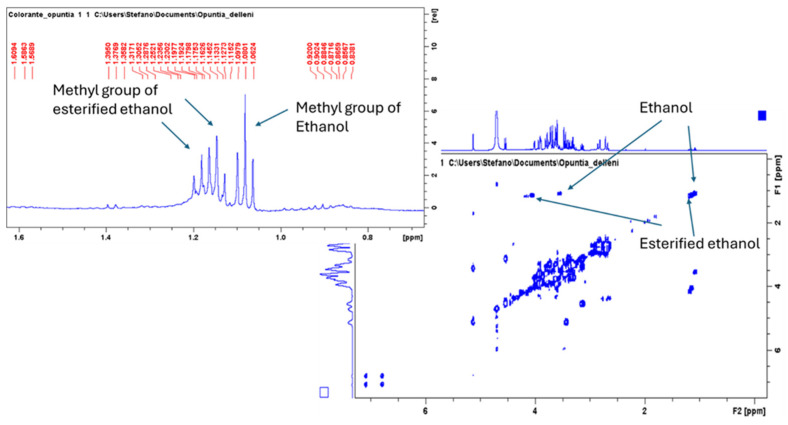
^1^H-NMR detail of the methyl groups and COSY spectrum of *Opuntia stricta* fruit pulp hydroalcoholic extract with the ethanol residual highlighted.

**Figure 4:**
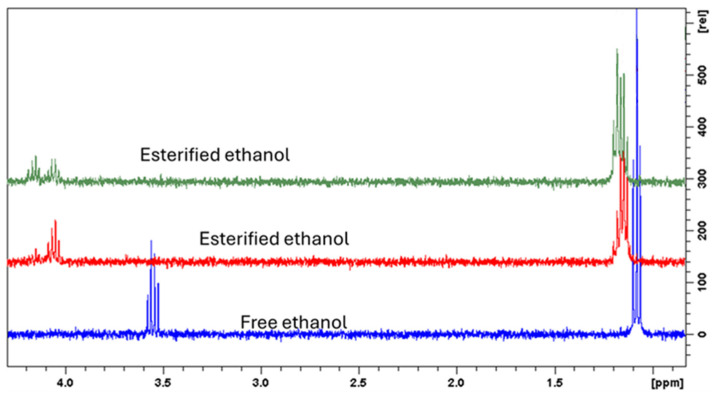
1D-TOCSY spectra of *Opuntia stricta* fruit pulp hydroalcoholic extract.

To establish the structure of this pigment, the extract was subjected to further nuclear magnetic resonance (NMR) analysis. First, the ^1^H-NMR shows the presence of two groups of signals ascribable as triplets at *δ* 1.08, 1.14, and 1.18 that are not observed in the freeze-dried fruit pulp sample. Furthermore, the spin coupling of these signals (*δ* 1.08, 1.14, and 1.18) was observed in the COSY spectrum showing coupling with *δ* 3.54 and with 4.05 and 4.17, respectively, suggesting that the first triplet is ascribable to ethanol while the other is ascribable to esterified ethanol residues (Figure 3). The acquisition of 1D-TOCSY spectra irradiating each of the methyl group signals allows us to observe the presence of three different ethyl esters, as shown in Figure 4.

A previous work indicates the thermal instability of betalain when heated, especially in water/ethanol mixtures [27].

The HR-MS in the ion trap shows that the *m*/*z* 318 appears to be double charged. For this reason, we can calculate the molecular weight of the derivative as 636, which corresponds to the tri ethanol ester of betanine. This is in agreement with the observation of the three ester ethanolic groups that were observed by ^1^H- COSY and 1D-TOCSY NMR. The MS^2^ spectrum originating from the fragmentation of *m*/*z* 318 shows intense ions at *m*/*z* 157 deriving from the loss of C_14_H_28_NO_7_; and further ions from *m*/*z* 157 are observed at *m*/*z* 111, 95, and 83. This peak corresponding to the triethyl ester of betanin appears only when the ethanol extract is concentrated and dried, so it is highly reasonable to postulate that a Fischer esterification occurs during extract concentration due to the presence of ethanol and the three carboxylic groups of betanin. As confirmation of the esterification, we also observed the NMR signals that we assigned to the ethyl ester groups, namely, the triplets at *δ* 1.08, 1.14, and 1.18 and the quartets at *δ* 4.05 and 4.17, supporting the esterification with ethanol.

The two peaks presenting molecular ions [M + H]^+^ at *m*/*z* 551 in MS^2^ show an intense prevalent peak at *m*/*z* 389 suggesting the loss of a hexose unit. The MS^3^ spectrum shows multiple peaks with a prevalent species at *m*/*z* 343 supporting the neutral loss of 46 Da and *m*/*z* 297 supporting the neutral loss of another 46 Da (Figure S2). These fragments can be explained by the loss of formic acid units from the betalain molecules with the formation of conjugated double bonds (Figure S3). One of the most abundant species is the one at *m*/*z* 150 that suggests the presence of only one nitrogen in the compound and a possible cyclization involving the acidic group to form a lacton. The MS^n^ spectra up to MS^5^ were collected, allowing for the identification of two betalain isomers. Based on the literature [28], the peaks were assigned to isobetanin (C-15 epimer of betanin) and betanin. The species observed at *m*/*z* 507 can be ascribed to the decarboxyl derivatives of betanin that have been previously reported [25]; the MS fragmentation shows the loss of a hexose unit and the further loss of formic acid or COOH, and thus the compounds are tentatively identified as possible decarboxyl betanin or isobetanin derivatives. Based on the published literature, the structure of the compound can be ascribed to 15-decarboxy or 17-decarboxy betanin [25]. Other ions are identified as piscidic acid (observed as [M − H]^−^ at *m*/*z* 255 in negative ion mode), and the flavonoids isorhamnetin-3-*O*-glucoside (observed as [M − H]^−^ at *m*/*z* 477 in negative ion mode) and isorhamnetin-3-*O*-rutinoside (observed as [M − H]^−^ at *m*/*z* 623 in negative ion mode) that were previously reported by Mata et al. [26]. The identified constituents are summarized in Table 2.

The phytochemical characterization of the freeze-dried fruit pulp and its hydroalcoholic extract showed the presence of betalain derivatives, piscidic acid, and flavonoid derivatives as previously reported by Gómez-López et al. [29]. The extract presents an unusual peak with ultraviolet–visible (UV–Vis) spectra comparable to those of the betalains, which can be ascribed to a tri ethanol ester of betanine, induced by the hydroalcoholic extraction step.

### 2.3. Gas Chromatography–Mass Spectrometry (GC-MS) of Opuntia Stricta Oil

The quantitative and qualitative analytical results of the studied *Opuntia stricta*’s seed fixed oil (OSS) by gas chromatography (GC) and gas chromatography–mass spectrometry (GC-MS) are shown in Table 3, where a total of 11 fatty acids, representing 87.8% of the total, have been identified. The polyunsaturated fatty acid, linoleic acid, was the most abundant compound with a percentage of 41.95%, followed by palmitic acid (19.32%). Significant amounts of stearic and oleic acids were also found with percentages of 10.84 and 8.03, respectively. A noteworthy content of unsaturated fatty acids at 54.19% was found.

Our results agree with those reported by Abdnim et al. [30], which found linoleic acid (36.59%) and palmitic acid (20.84%) as the main abundant fatty acids of *Opuntia ficus-indica* seed oil. Moroccan *Opuntia ficus-indica* and *Opuntia dillenii* seed oils were investigated for their fatty acids [31]. A higher concentration of linoleic acids was found with percentages of 58.79 and 79.83% for *Opuntia ficus-indica* and *Opuntia dillenii* oils, respectively. Palmitic acid was less abundant, as was stearic acid.

A higher linoleic acid content (71.43%) was found by El Mannoubi et al. [32] in Tunisian *Opuntia stricta* oil. A different pattern was observed for oleic (12.24%) and palmitic acids (10.81%). A higher linoleic acid content was found in Iraqi *O. dillenii* seed oil with a percentage of 72.9%. Palmitic acid (15.12%) and stearic acid (7.51%) were also found as the main fatty acids in the seeds [33].

Higher levels of linoleic acid (70%) were detected in *Opuntia stricta* seed oil from Africa. In this oil, the authors also identified palmitic (12.5%) and stearic (12.3%) acids, with a high unsaturation level (83%) [24].

### 2.4. Antioxidant Activity

Antioxidant molecules provide a valid contribution to the management of oxidative stress, associated with various chronic diseases. The scientific community recommends a multi-analytical approach that can highlight the natural extracts/compounds with greater activity [34]. In this context, we have applied different tests to investigate the antioxidant potential of the *Opuntia stricta* samples.

The analysis of the results evidenced that, referring to the DPPH data, the freeze-dried sample exhibited the highest radical-scavenging potential with an IC_50_ value of 23.77 μg/mL followed by the hydroalcoholic extract of the fruit pulp (IC_50_ value of 44.94 μg/mL) (Table 4). A comparable ABTS^•+^ radical-scavenging potential was observed when the freeze-dried fruit pulp (OSF) and its hydroalcoholic extract (OSC) were tested (IC_50_ values of 13.24 and 14.82 μg/mL for OSC and OSF, respectively). Unlike in the DPPH, where the oil activity appeared very limited, in the ABTS tests, an IC_50_ value of 34.40 μg/mL was found. The main difference between the DPPH and ABTS tests lie in the type of free radical used and the testing conditions. When an antioxidant interacts with DPPH, it reduces the DPPH by donating an electron or a hydrogen atom, changing the solution’s color from purple to a clear or light yellow. The 2,2-Diphenyl-1-picrylhydrazyl (DPPH) test is primarily used in aqueous solutions; it is sensitive to pH changes or the solubility of the tested compounds. 2,2-Azinobis (3-ethylbenzothiazoline-6-sulfonic acid) (ABTS^•+^) is a cationic radical generated in the lab by the oxidation of ABTS. Similarly, antioxidants neutralize the ABTS radical by donating an electron or a hydrogen atom, changing the color from green-blue to colorless. ABTS is more versatile than DPPH, as the test can be performed in aqueous or organic solvents. It is useful for testing both lipid-soluble and water-soluble antioxidant compounds and offers higher sensitivity.

Lipids are significant nutrients for humans and help many functional and regulatory activities in the human body, including the signal transduction of synaptic plasticity, and myelination. Lipids are also involved in the structural developments of the human body [35]. For this reason, it is important to investigate the ability of the extract to counteract lipid oxidation. Therefore, we have used the *β*-carotene bleaching test that mimics lipid peroxidation. In this test, the *Opuntia stricta* hydroalcoholic extract exhibited the highest protection from lipid peroxidation with an IC_50_ value of 23.15 μg/mL, followed by the freeze-dried fruit pulp (IC_50_ value of 25.78 μg/mL). The data show that the fixed oil was significantly less powerful (IC_50_ value of 75.99 μg/mL).

The ferric reducing antioxidant power (FRAP) test was also applied with the aim of investigating the ability of the extract to counteract Fe(II) oxidation. In this case, both fruit samples exhibited higher FRAP values (89.91 and 76.89 μMFe (II)/g, respectively) than those found for the butylated hydroxytoluene, used as a positive control. A great variability in antioxidant activity results are found in the literature.

Previously, Katanić et al. [36] investigated the antioxidant potential of skin, seeds, and juice from *Opuntia dillenii* collected in different areas of Morocco. Juice ethanol extract exhibited IC_50_ values of 14.5 and 16.6 μg/mL against the DPPH and the ABTS radical, respectively. These values agree with those reported by Ghazi et al. [37], who found an IC_50_ value of 8.18 μg/mL against DPPH. Moreover, the same research group found an IC_50_ value higher than that of ascorbic acid when *Opuntia dillenii* syn. *stricta* seed oil was examined using the DPPH scavenging assay (27.21 vs. 16.56 μL/mL). Both data are higher than those reported in our investigation. *Opuntia stricta* dried fruits are rich in betalains such as betanin and isobetanin. In particular, betanin exerted a radical-scavenging activity against DPPH in a dose-dependent manner [38]. Cai et al. [39] demonstrated that betanin exerted a DPPH radical-scavenging effect with ‘half-maximal effective concentration (EC_50_) values of 4.88 μM, whereas a value of 4.89 μM was found for the C_15_ epimer isobetanin. Recently, Sakihama et al. [40] reported that a difference in DPPH radical-scavenging potential was found when a natural betanin and a commercial one were tested, with IC_50_ values of 49.18 and 55.8 mM, respectively. A DPPH radical-scavenging potential of about 1000 times higher (65.8 μM) was found by Muramatsu et al. [41]. Betanin exerts its antioxidant activities due to its electron donor capacity, the bond dissociation energy, and the ionization potential of the molecule [42].

Moreover, both compounds are able to exert ferric reducing ability power (FRAP) with percentages of 56.82 and 15.96% for betanin and isobetanin, respectively.

Moreover, betanin was effective in reducing the ABTS^+^ radical and was able to donate electrons and reduce the ferric ion of the tripyridyltriazine complex (Fe^3+^-TPTZ) [32]. In fact, Tsai et al. [43] reported that both betalains are able to exert ferric reducing ability power (FRAP) with percentages of 56.82 and 15.96% for betanin and isobetanin, respectively. Betanin, the main pigment of red beets, has a molecular origin that results in its exceptionally high free radical-scavenging activity [42].

This phytochemical was able to inhibit linoleic acid peroxidation with an IC_50_ value of 0.4 μM. This value is lower than those found for a well-known antioxidant, R-tocopherol (5 μM) [44].

Betanin is the only betalain approved for applications in the pharmaceutical and food industries. Its high antioxidant power was retained even after small intestine digestion, as reported by Vieira Teixeira da Silva et al. [45], where almost 54% of the originally administered amount was found unmodified. *Opuntia stricta* fruits were also rich in isorhamnetin. This flavonol exhibited IC_50_ values of 6.67, 14.54, and 24.61 μM in the inhibition of lipid peroxidation, ABTS, and DPPH tests [46].

### 2.5. Carbohydrate-Hydrolizing Enzymes and Lipase Inhibitory Activities

The samples were also tested in terms of their inhibitory capacity towards enzymes involved in carbohydrate digestion, such as α-amylase and α-glucosidase. The hypolipidemic activity was instead evaluated through the inhibition of pancreatic lipase (Table 5). In particular, α-amylase hydrolyzes 1,4-glycosidic linkages of polysaccharides (such as starch and glycogen) to disaccharides, whereas α-glucosidase catalyzes the conversion of disaccharides into monosaccharides; for these reasons, both enzymes are involved at the postprandial glycemia level. Inhibitors of these enzymes delay carbohydrate digestion and are useful in the control of hyperglycemia.

Generally, the α-glucosidase enzyme resulted in more sensitivity to the activity of the *Opuntia stricta* samples. In particular, the freeze-dried fruit sample (OSF) showed the highest inhibitory activity against both α-amylase and α-glucosidase with IC_50_ values of 105.43 and 130.37 μg/mL, respectively). A significant α-glucosidase inhibitory effect was also found with hydroalcoholic extract (OSC, IC_50_ value of 158.51 μg/mL). A great variability in lipase inhibition was observed. The freeze-dried pulp proved to have an inhibitory power that was superior, albeit slightly, to the positive control orlistat with IC_50_ values of 33.54 and 37.44 μg/mL, respectively.

A perusal analysis of the literature revealed that *Opuntia dillenii* seeds, juice, and peel ethanolic extracts can inhibit both carbohydrate-hydrolyzing enzymes in a dose-dependent manner [10]. Results evidenced that *Opuntia dillenii* peel extracts exhibited the greatest effect, followed by fruit pulp and seed extracts. Moreover, Bouhrim et al. [12] demonstrated that Moroccan *Opuntia dillenii* seed oil significantly inhibited both pancreatic α-amylase and intestinal α-glucosidase with IC_50_ values of 810 and 278 µg/mL, respectively.

In vivo studies evidenced that the oil significantly attenuated postprandial hyperglycemia in both normal and streptozotocin (STZ)-diabetic rats.

Previously, Kunyanga et al. [24] investigated the effect of Kenyan *O. stricta* fruit extract. A more significant activity against α-glucosidase compared to *α*-amylase was detected. The ability of *Opuntia* phytochemicals to inhibit pancreatic lipase was also evidenced by Mihri et al. [47], who demonstrated the pancreatic lipase inhibitory activity of Tunisian *Opuntia ficus-indica* and *Opuntia stricta* flowers’ essential oils and found IC_50_ values of 100.53 and 120.96 µg/mL, respectively. A moderate activity against α-amylase was also observed with IC_50_ values of 89.37 and 100.63 µg/mL for *Opuntia ficus-indica* and *Opuntia stricta*, respectively.

Betanin, identified in both the freeze-dried and hydroalcoholic extract of *Opuntia stricta* fruits, exhibited a dose-dependent α-amylase and α-glucosidase inhibitory activities with IC_50_ values of 1.75 and 2.85 μg/mL, respectively [48].

Moreover, betanin has been reported to have potential anti-obesity activity by inhibiting lipogenesis and enhancing lipolysis and fatty acid oxidation [49].

Recently, Serrano-Sandoval et al. [50] stated from the evidence that *Opuntia stricta* var. *dillenii* fruit has been recognized for its effects on hepatic steatosis, identifying one of the main compounds responsible for the activity, as well as betalains such as betanin and isobetanin. All these phytochemicals are identified in our samples. Both the freeze-dried fruit and its extract contain isorhamnetin. Isorhamnetin-3-*O*-rutinoside and isorhamnetin-3-*O*-glucoside, both identified in our sample, inhibited the α-amylase enzyme with IC_50_ values of 0.129 and 0.619 mM [51]. More recently, a molecular docking study evidenced that this phytochemical exerts α-amylase inhibitory activity with an inhibitory potential of −8.5 Kcal/mol that resulted in lower values as compared with those of acarbose, a commonly prescribed drug (−7.1 Kcal/mol) [52]. At the same time, it is important to note that isorhamnetin did not exert α-glucosidase inhibitory activity and for this reason, this compound does not contribute to the activity shown by the *Opuntia stricta* fruit sample and extract [53].

Referring to anti-obesity activity, isorhamnetin-3-*O*-rutinoside exhibited a pancreatic lipase inhibitory effect with an IC_50_ value of 237.87 µg/mL [54]. Moreover, in vitro and in vivo studies demonstrated that isorhamnetin and its glycosides are effective as anti-obesity agents. A perusal analysis of the literature highlights, in addition to the previously mentioned glycemic control through the inhibition of α-amylase, a possible reduction in both lipogenesis and fatty acid absorption [55].

## 3. Materials and Methods

### 3.1. Plant Materials and Extraction Procedure

**Figure 5:**
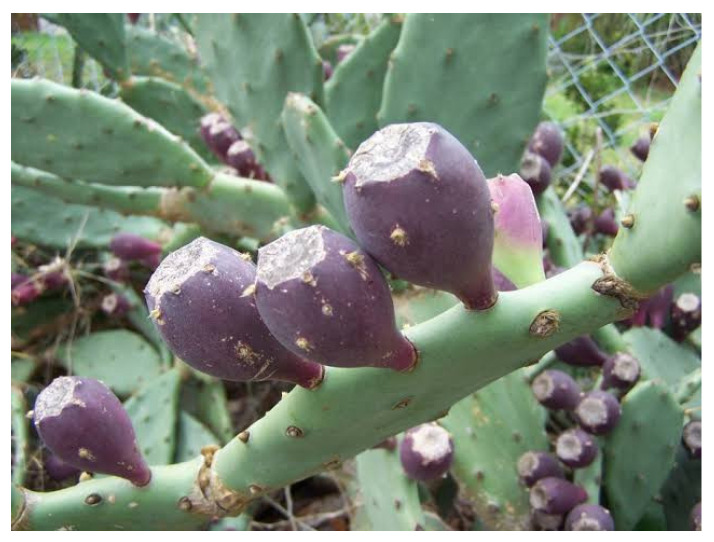
*Opuntia stricta* fruits.

*Opuntia stricta* (Haw.) fruits were collected in Barcarello, Sferracavallo, Palermo, (Sicily, Italy) (38°12′36″ N, 13°16′56″ E, 2 m.s.l.) during April 2024 (Figure 5). Authentication was performed by Prof. Francesco Sottile, and a voucher specimen was deposited in the University of Palermo (reference number PAL113519).

The fruits (1 kg) were peeled, and the seeds (27 g) were mechanically removed. The resulting pulp (700 g) was blended with distilled water and filtered to give 520 g. One hundred g of the sample was freeze-dried to give 8 g of dry material (OSF). Fifty g of the freeze-dried sample was mixed with a hydroalcoholic solution (8:2 *v*/*v*) at pH 5, to precipitate the mucilage. The solution was evaporated to give 2.5 g of hydroalcoholic extract (OSC).

Twenty grams of *Opuntia stricta* fruit seeds was grinded and extracted with *n*-hexane for one week (200 mL × 3-times) to give, after evaporation, 500 mg of fixed oil (OSS).

### 3.2. Gas Chromatography–Mass Spectrometry (GC-MS) Analysis

*Opuntia stricta* fixed oil (OSS) was analyzed by a Hewlett-Packard gas chromatograph (Agilent, Milan, Italy) equipped with a non-polar HP-5 capillary column (30 m × 0.25 mm, 0.25 μm), associated with a Hewlett-Packard mass spectrometer (Agilent, Milan, Italy). The following operating conditions have been applied: electronic impact (EI, 70 eV); isotherm at 50 °C for 5 min, then from 50 to 250 °C with an increase of 5 °C/min, and finally isotherm at 250 °C for 10 min. Compound identification was carried out by using mass spectral data (Wiley 138 library and referring to the spectral data of pure standards and compounds known in the literature).

### 3.3. Total Phenolic, Flavonoid, and Carotenoid Compounds

The total phenol content (TPC) was estimated spectrophotometrically by using the Folin–Ciocalteu method [56]. Results were expressed as mg of chlorogenic acid equivalents (CAE)/g of fresh weight (FW).

The total flavonoid content (TFC) was determined spectrophotometrically using a method based on the formation of a flavonoid–aluminum complex [57]. The total flavonoid content was expressed as mg of quercetin equivalents (QE)/g FW.

### 3.4. Phytochemical Analysis of Opuntia Stricta Fruit Pulp Samples

The analysis of pigments was obtained using a UV–Visible spectrophotometer (Shimadzu UV–Visible 160 A, Tokyo, Japan) with the method reported by Khatabi et al. with small modifications [58]. One hundred mg of each sample was suspended in 25 mL of ethanol/water (8:2 *v*/*v*) and homogenized by magnetic stirring. The mixture was then filtered through a nylon filter (0.45 µm). The measurements were made using a quartz cell of 1 cm. The quantities of pigment extracts were evaluated by spectrophotometry according to Lambert–Beer’s law. The results were expressed as mg pigment per % *m*/*m* of extract.

The analysis of the samples was performed on a 1260 Agilent chromatograph (Santa Clara, CA, USA) coupled with a diode array (DAD) and a mass spectrometer, an ion trap MS 500 Varian (Santa Clara, CA, USA). The column was an Agilent C18 XDB 3 × 150 mm, 3.5 micron (Santa Clara, CA, USA). The flow occurred after the column was split into the DAD detector and ion trap. The used mobile phase was water 1% formic acid (A), acetonitrile (B), and methanol (C) with the following gradient: 0 min 100%:0%:0% A:B:C, 14 min 80%:0%:20%; 20 min 55%:0%:45%; 45 min 0%:20%:80%, 50 min 0%:20%:80%. The DAD detector was set at 254, 312, 540, 280, 350 nm. The mass spectrometer was equipped with an ESI ion source, working both in the negative and positive modes. Fifty mg amounts of both the fruit pulp samples were suspended in water 1% formic acid and sonicated. The liquid was centrifuged, and the supernatant was used for the analysis.

The fruit pulp samples were analyzed by NMR dissolving 50 mg in 1.5 mL of deuterated water. The suspension was centrifuged to separate any remaining chloroform layer and transferred to the NMR tube. A NMR Bruker Avance III was used to perform the 1D and 2D experiments, namely, ^1^H, the 1D TOCSY experiment, and COSY.

### 3.5. Evaluation of Opuntia Stricta Samples’ Antioxidant Activities

The multi-analytical approach was used for *Opuntia stricta* extracts’ antioxidant activity evaluation. The 1,1-diphenyl-2-picrylhydrazyl (DPPH) and the 2,2-azino-bis (3-ethylbenzothiazoline-6-sulfonic acid) (ABTS) radical activities were performed as previously reported [57]. For the 2,2-azino-bis (3-ethylbenzothiazoline-6-sulfonic acid) (ABTS) assay, a solution of ABTS was prepared and left in the dark for 12 h. A mixture of samples at different concentrations (1–400 µg/mL) and the diluted ABTS solution was formulated and, after 6 min, the absorbance was measured (734 nm). Ascorbic acid was used as the positive control in both radical-scavenging assays. Briefly, a solution of DPPH (1.0 × 10^−4^ M) was mixed with the sample (at concentrations in the range of 1–1000 µg/mL). The absorbance was read at 517 nm. The ferric reducing antioxidant power (FRAP) was executed as previously reported [57]. The FRAP reagent was prepared by mixing 10 mM of a 2,4,6-tripyridyl-s-triazine (TPTZ) solution with HCl, acetate buffer (pH 3.6), and 20 mM FeCl_3_. A mixture of the extract (2.5 mg/mL), water, and FRAP reagent was prepared and incubated for 30 min at 25 °C. The absorbance was measured at 595 nm. The value was expressed as μM Fe(II)/g. Butylated hydroxytoluene (BHT) was used as a positive control.

The protection of lipid peroxidation was tested by the *β*-carotene bleaching assay [57]. An emulsion of *β*-carotene, Tween 20, and linoleic acid was mixed with the sample (at a concentration in the range of 5–100 μg/mL). The absorbance was read at λ = 470 nm after 30 min of incubation (at 45 °C). Propyl gallate was used as a positive control.

### 3.6. Evaluation of α-Amylase, α-Glucosidase, and Lipase Inhibitory Activities

For the inhibition of pancreatic lipase enzymes, the method previously described [59] was applied. In this assay, a mixture of the samples, 4-nitrophenyl octanoate (NPC), Tris-HCl buffer (pH 8.5), and enzyme solution, was added in a 96-well plate and incubated at 37 °C for 30 min. The absorbance was determined at λ = 412 nm and orlistat was used as a positive control.

In the α-glucosidase inhibitory activity test, a maltose solution, an enzyme (EC 3.2.1.20) solution, and an *o*-dianisidine solution were prepared and mixed. This mixture was left to incubate at 37 °C for 30 min. Then, perchloric acid was added. The supernatant was collected and mixed with DIAN and PGO, and left to incubate at 37 °C for 30 min. The absorbance was read at 500 nm and acarbose was used as a positive control in both tests.

For the inhibition of α-amylase and α-glucosidase enzymes, the method of Loizzo et al. [40] was applied. In the *α*-amylase inhibitory assay, a starch solution of enzymes (EC 3.2.1.1) and colorimetric reagent was prepared. Both the control and the extract were added to the starch solution and left to react with the enzymes. The absorbance was read at 540 nm. The results are expressed as 50% inhibitory concentration (IC_50_).

### 3.7. Statistical Analysis

Experiments were carried out in triplicate. Prism GraphPad Prism version 4.0 for Windows (GraphPad Software, San Diego, CA, USA) was used to calculate the concentration, giving 50% inhibition (IC_50_). Tukey’s test was used to determine any significant differences in the chemical parameters among the investigated samples.

## 4. Conclusions

The *Opuntia stricta* freeze-dried fruit pulp and hydroalcoholic extract contain betalains, piscidic acid, and isorhamnetin derivatives as the main abundant compounds. The sample obtained by extraction with hydroalcoholic solution presents a betaine derivative that probably is obtained during the extraction and drying procedure. The fatty acid profile of the fixed oil showed a high content of linoleic and palmitic acids. Antioxidant activity was assessed using a multi-target approach using the ABTS, DPPH, FRAP, and *β*-carotene bleaching tests. The freeze-dried fruit pulp resulted in the most active in DPPH radical-scavenging test, whereas a comparable activity between the freeze-dried fruit sample and its hydroalcoholic extract was observed in the ABTS and *β*-carotene bleaching tests. The *Opuntia stricta* samples were assessed by carbohydrate-hydrolyzing enzyme and lipase inhibitory tests, where the freeze-dried fruit pulp sample exerted the highest activity. Collectively, our results evidenced that *Opuntia stricta* is a food matrix with promising functional properties with reference to the hypoglycemic and anti-obesity effect. These activities are strictly related to the betalain and flavonoid content.

## Figures and Tables

**Table 1:** Total phytochemical content of *Opuntia stricta* freeze-dried fruit pulp (OSF) and its hydroalcoholic extract (OSC).

Samples	Total Phenol Content (TPC) (mg of Chlorogenic Acid Equivalent (CAE)/g Fresh Weight (FW))	Total Flavonoid Content (TFC) (mg of Quercetin Equivalent (QE)/g Fresh Weight (FW))
OSF	546.09 ± 11.34 ^a^	276.02 ± 6.31 ^a^
OSC	458.12 ± 10.65 ^b^	125.23 ± 5.20 ^b^
*Sign.*	***	***

OSF: *Opuntia stricta* fruit pulp; OSC: *Opuntia stricta* hydroalcoholic fruit pulp extract; Data are expressed as means ± S.D. (*n* = 3). Differences letters (a and b) were evaluated by a one-way analysis of variance (ANOVA) test completed with a multi-comparison Tukey’s test. *** *p* < 0.01.

**Table 2:** Identified compounds in *Opuntia stricta*.

Retention Time	[M + H]^+^	Fragments	Identification	Freeze-Dried Fruit Pulp Sample	Freeze-Dried Fruit Pulp Hydroalcoholic Extract
15.3	551	MS2: 389 MS3(389):345 343 301 246 194 150	Betanin	√	√
16.7	551	MS2: 389 MS3(389):345 343 301 246 194 150	Isobetanin	√	√
11.9	507	MS2: 345 MS3(345) 301 314 150	Decarboxy betanin	√	
12.9	507	MS2: 345 MS3(345) 301 314 150	Decarboxy betanin	√	
13.9	507	MS2: 345 MS3(345) 301 314 150	Decarboxy betanin	√	
**Retention time**	**[M + 2H]^2+^**	**Fragments**	**Identification**	**Freeze-dried sample**	**Hydroalcoholic extract**
12.3	318	157	Triethanol-betanin		√
**Retention time**	**[M − H]** ^−^	**Fragments**	**Identification**	**Freeze-dried sample**	**Hydroalcoholic extract**
12.0	255		Piscidic acid	√	√
26.7	623	315	Isorhamnetin-3-*O*-rutinoside	√	√
26.4	477	315	Isorhamnetin-3-*O*-glucoside	√	√

**Table 3:** The main fatty acids of *Opuntia stricta*’s seed oil.

Fatty Acids	%
Myristic acid (C14:0)	0.25 ± 0.01
Palmitoleic acid (C16:1)	1.55 ± 0.65
Palmitic acid (C16:0)	19.32 ± 1.28
Linoleic acid (C18:2)	41.95 ± 2.66
Oleic acid (C18:1)	8.03 ± 1.33
Stearic acid (C18:0)	10.84 ± 1.85
Gondoic acid (C20:1)	2.01 ± 0.07
Arachidic acid (C20:0)	1.55 ± 0.65
Erucid acid (C22:1)	0.65 ± 0.03
Behenic acid (C22:0)	1.08 ± 0.08
Lignoceric acid (C24:0)	0.58 ± 0.02
**Total identified**	**87.81**
**SFA**	**33.62**
**UFA**	**54.19**
**UFA/SFA**	**1.61**
**Oleic/linoleic**	**0.20**

Data are reported as mean ± standard deviation (*n* = 3). Saturated fatty acids (SFA); unsaturated fatty acids (UFA); unsaturation ratio (UFA/SFA).

**Table 4:** Antioxidant activity of *Opuntia stricta* samples.

Sample	DPPH IC_50_ (µg/mL)	ABTS IC_50_ (µg/mL)	*β*-Carotene Bleaching Test IC_50_ (µg/mL)	FRAP ^ μMFe (II)/g
OSF	23.77 ± 1.26 ^a^	14.82 ± 1.33 ^b^	25.78 ± 1.23 ^a^	89.91 ± 3.26 ^a^
OSC	44.94 ± 2.65 ^b^	13.24 ± 1.56 ^a^	23.15 ± 1.44 ^b^	76.89 ± 3.12 ^b^
OSS	57.91 ± 2.48 ^c^	32.40 ± 2.12 ^b^	75.99 ± 3.45 ^c^	23.15 ± 1.23 ^c^
*Sign.*	***	***	***	***
Ascorbic acid	5.10 ± 0.98	1.71 ± 0.86		
Propyl gallate			0.09 ± 0.00	
BHT				63.02 ± 11.45

OSF: *Opuntia stricta* fruit pulp; OSC: *Opuntia stricta* hydroalcoholic fruit pulp extract; OSS: *Opuntia stricta* seed fixed oil. Data are expressed as means ± S.D. (*n* = 3). Ascorbic acid was used as a positive control in both the DPPH and ABTS test; butylated hydroxytoluene (BHT) and propyl gallate were used in the FRAP and *β*-carotene bleaching tests, respectively. ^^^ at concentration 2.5 mg/mL. Differences letters (a, b, and c) were evaluated by a one-way analysis of variance (ANOVA) test completed with a multi-comparison Tukey’s test. *** *p* < 0.01.

**Table 5:** Carbohydrate-hydrolyzing enzymes and lipase inhibitory effects (IC_50_ (µg/mL) of *Opuntia stricta* samples.

Sample	α-Amylase	α-Glucosidase	Pancreatic lipase
OSF	105.43 ± 3.77 ^a^	130.37 ± 3.85 ^a^	33.54 ± 3.92 ^a^
OSC	415.50 ± 3.56 ^b^	158.51 ± 3.77 ^b^	81.77 ± 3.44 ^b^
OSS	459.44 ± 3.56 ^b^	169.94 ± 3.81 ^c^	100.45 ± 3.44 ^c^
*Sign.*	***	***	***
Acarbose	50.18 ± 1.32	35.57 ± 1.99	
Orlistat			37.44 ± 1.08

OSF: *Opuntia stricta* fruit pulp; OSC: *Opuntia stricta* hydroalcoholic fruit pulp extract; OSS: *Opuntia stricta* seed fixed oil. Data are expressed as means ± S.D. (*n* = 3). Acarbose was used as a positive control in both the a-amylase and a-glucosidase tests, whereas orlistat was used in the lipase inhibitory activity test. Differences letters (a, b, and c) were evaluated by a one-way analysis of variance (ANOVA) test completed with a multi-comparison Tukey’s test. *** *p* < 0.01.

## Data Availability

The raw data supporting the conclusions of this article will be made available by the authors on request.

## Supplementary Materials

The following supporting information can be downloaded at https://www.mdpi.com/article/10.3390/molecules30071580/s1, Figure S1: LC-MS chromatograms of the ions [M + H]^+^ 551 and 507 of the OSF extract; Figure S2: Mass spectra of ion 551 *m*/*z*; Figure S3: Proposed mass fragmentation pathway of the ion at *m*/*z* 551.

## References

[1] Vilà M., Burriel J.A., Pino J., Chamizo J., Llach E., Porterias M., Vives M. (2003). Association between *Opuntia* species invasion and changes in land-cover in the Mediterranean region. Glob. Change Biol..

[2] Stintzing F.C., Carle R. (2005). Cactus stems (*Opuntia* spp.): A review on their chemistry, technology, and uses. Mol. Nutr. Food Res..

[3] D’Agostino G., Merra R., Badalamenti N., Lazzara G., Bruno M., Sottile F. (2023). *Opuntia ficus-indica* (L.) Mill. and *Opuntia stricta* (Haw.) Haw. Mucilage-Based Painting Binders for Conservation of Cultural Heritage. Sustainability.

[4] Madrigal-Santillán E., Portillo-Reyes J., Madrigal-Bujaidar E., Sánchez-Gutiérrez M., Izquierdo-Vega J.A., Izquierdo-Vega J., Delgado-Olivares L., Vargas-Mendoza N., Álvarez-González I., Morales-González Á. (2022). *Opuntia* spp. in Human Health: A Comprehensive Summary on Its Pharmacological, Therapeutic and Preventive Properties. Part 2. Plants.

[5] Hultine K.R., Hernández-Hernández T., Williams D.G., Albeke S.E., Tran N., Puente R., Larios E. (2023). Global change impacts on cacti (Cactaceae): Current threats, challenges and conservation solutions. Ann. Bot..

[6] Chandrasekaran P., Weiskirchen R. (2024). The Role of Obesity in Type 2 Diabetes Mellitus—An Overview. Int. J. Mol. Sci..

[7] ISTAT (2021). Aspetti Della Vita Quotidiana. Fattori di Rischio per la Salute: Fumo, Obesità, Alcol e Sedentarietà.

[8] Liu X., Zeng X., Liu W., Lu Y., Cheng J., Chen Y. (2021). An overview of dietary supplements on obesity and type 2 diabetes: Efficacy and mechanisms. Curr. Drug. Metab..

[9] Caturano A., D’Angelo M., Mormone A., Russo V., Mollica M.P., Salvatore T., Galiero R., Rinaldi L., Vetrano E., Marfella R. (2023). Oxidative stress in type 2 diabetes: Impacts from pathogenesis to lifestyle modifications. Curr. Issues Mol. Biol..

[10] Loukili E.H., Abrigach F., Bouhrim M., Bnouham M., Fauconnier M.L., Ramdani M. (2021). Chemical composition and physicochemical analysis of *Opuntia dilleniii* extracts grown in Morocco. J. Chem..

[11] Loukili E.H., Merzouki M., Taibi M., Elbouzidi A., Hammouti B., Kumar Yadav K., Khalid M., Addi M., Ramdani M., Kumar P. (2024). Phytochemical, biological, and nutritional properties of the prickly pear, *Opuntia dilleniii*: A review. Saudi Pharm. J..

[12] Bouhrim M., Ouassou H., Boutahiri S., Daoudi N.E., Mechchate H., Gressier B., Eto B., Imtara H., Alotaibi A.A., Al-Zharani M. (2021). *Opuntia dilleniiid* (Ker Gawl.) Haw., seeds oil antidiabetic potential using in vivo, in vitro, in situ, and ex vivo approaches to reveal its underlying mechanism of action. Molecules.

[13] Montalbano G., La Rosa M. (2010). Flora della Sicilia.

[14] Intharuksa A., Kuljarusnont S., Sasaki Y., Tungmunnithum D. (2024). Flavonoids and Other Polyphenols: Bioactive Molecules from Traditional Medicine Recipes/Medicinal Plants and Their Potential for Phytopharmaceutical and Medical Application. Molecules.

[15] Ćorković I., Gašo-Sokač D., Pichler A., Šimunović J., Kopjar M. (2022). Dietary Polyphenols as Natural Inhibitors of α-Amylase and α-Glucosidase. Life.

[16] Buchholz T., Melzig M.F. (2015). Polyphenolic Compounds as Pancreatic Lipase Inhibitors. Planta Med..

[17] Chahdoura H., Mzoughi Z., Ellouze I., Generalić Mekinić I., Čmiková N., El Bok S., Majdoub H., Ben Hsouna A., Ben Saad R., Mnif W. (2024). *Opuntia* species: A comprehensive review of chemical composition and bio-pharmacological potential with contemporary applications. S. Afr. J. Bot..

[18] Zeghbib W., Boudjouan F., Vasconcelos V., Lopes G. (2022). Phenolic Compounds’ Occurrence in *Opuntia* Species and Their Role in the Inflammatory Process: A Review. Molecules.

[19] Shirazinia R., Golabchifar A.A., Rahimi V.B., Jamshidian A., Samzadeh-Kermani A., Hasanein P., Hajinezhad M., Askari V.R. (2021). Protective Effect of *Opuntia dilleniii* Haw Fruit Against Lead Acetate-Induced Hepatotoxicity: In Vitro and In Vivo Studies. Evid. Based Complement. Alternat. Med..

[20] Marhri A., Rbah Y., Allay A., Boumediene M., Tikent A., Benmoumen A., Melhaoui R., Elamrani A., Abid M., Addi M. (2024). Comparative Analysis of Antioxidant Potency and Phenolic Compounds in Fruit Peel of *Opuntia robusta*, *Opuntia dilleniii*, and *Opuntia ficus-indica* Using HPLC-DAD Profiling. J. Food Qual..

[21] Reis C.M.G., Gouveia C., Vitorino M.C., Gazarini L.C., Ribeiro M.M., Peres F. (2017). Bioactive compounds and morphology In *Opuntia* spp. fruits from *Portuguese ecotypes*. Bulg. J. Agric. Sci..

[22] Díaz-Medina E.M., Rodríguez-Rodríguez E.M., Díaz-Romero C. (2007). Chemical characterization of *Opuntia dilleniii* and *Opuntia ficus-indica* fruits. Food Chem..

[23] Moussa-Ayoub T.E., El-Samahy S.K., Rohn S., Kroh L.W. (2011). Flavonols, betacyanins content and antioxidant activity of Cactus *Opuntia macrorhiza* fruits. Food Res. Int..

[24] Kunyanga C.N., Imungi J.K., Vadivel V. (2014). Nutritional quality, phytochemical composition and health protective effects of an under-utilized prickly cactus fruit (*Opuntia stricta* Haw.) collected from Kenya. Afr. J. Food Agric. Develop..

[25] Florian C.S., Jürgen C., Iris K., Uwe B., Reinhold C. (2004). Structural investigations on betacyanin pigments by LC NMR and 2D NMR spectroscopy. Phytochemistry.

[26] Mata A., Ferreira J.P., Semedo C., Serra T., Duarte C.M., Bronze M.R. (2016). Contribution to the characterization of *Opuntia* spp. juices by LC-DAD-ESI-MS/MS. Food Chem..

[27] Otalora C.M., Bonifazi E., Fissore E.N., Basanta F., Gerschenson L.N. (2020). Thermal Stability of Betalains in By-Products of the Blanching and Cutting of *Beta vulgaris* L. var *conditiva*. Pol. J. Food Nutr. Sci..

[28] Cai Y., Sun M., Corke H. (2005). HPLC Characterization of Betalains from Plants in the Amaranthaceae. J. Chromatogr. Sci..

[29] Gómez-López I., Mendiola J.A., Portillo M.P., Cano M.P. (2022). Pressurized green liquid extraction of betalains and phenolic compounds from *Opuntia stricta* var. *dilleniii* whole fruit: Process optimization and biological activities of green extracts. Innov. Food Sci. Emerg. Technol..

[30] Abdnim R., Lafdil F.Z., Elrherabi A., El Fadili M., Kandsi F., Benayad O., Legssyer A., Ziyyat A., Mekhfi H., Bnouham M. (2024). Fatty acids characterisation by GC-MS, antiglycation effect at multiple stages and protection of erythrocytes cells from oxidative damage induced by glycation of albumin of *Opuntia ficus-indica* (L.) Mill seed oil cultivated in Eastern Morocco: Experimental and computational approaches. J. Ethnopharmacol..

[31] Ghazi Z., Ramdani M., Fauconnier M.L., El Mahi B., Cheikh R. (2013). Fatty acids Sterols and Vitamin E composition of seed oil of *Opuntia ficus-indica* and *Opuntia dilleniii* from Morocco. J. Mater. Environ. Sci..

[32] El Mannoubi I. (2021). Tunisian *Opuntia stricta* seed oil: Extraction, characterization, and prediction of fatty acid methyl ester properties as biodiesel fuel. Chem. Nat. Compd..

[33] Alsaad A.J.A., Altemimi A.B., Aziz S.N., Lakhssassi N. (2019). Extraction and Identification of Cactus *Opuntia dilleniii* Seed Oil and Its added Value for Human Health Benefits. Pharmacogn. J..

[34] Flieger J., Flieger W., Baj J., Maciejewski R. (2021). Antioxidants: Classification, Natural Sources, Activity/Capacity Measurements, and Usefulness for the Synthesis of Nanoparticles. Materials.

[35] Goodman B.E. (2010). Insights into digestion and absorption of major nutrients in humans. Adv. Physiol. Educ..

[36] Katanić J., Yousfi F., Caruso M.C., Matić S., Monti D.M., Loukili E.H., Boroja T., Mihailović V., Galgano F., Imbimbo P. (2019). Characterization of bioactivity and phytochemical composition with toxicity studies of different *Opuntia dilleniii* extracts from Morocco. Food Biosci..

[37] Ghazi Z., Ramdani M., Tahri M., Rmili R., Elmsellem H., El Mahi B., Fauconnier M.L. (2015). Chemical composition and antioxidant activity of seeds oils and fruit juice of *Opuntia ficus indica* and *Opuntia dilleniii* from Morocco. J. Mater..

[38] Esatbeyoglu T., Wagner A.E., Motafakkerazad R., Nakajima Y., Matsugo S., Rimbach G. (2014). Free radical scavenging and antioxidant activity of betanin: Electron spin resonance spectroscopy studies and studies in cultured cells. Food Chem. Toxicol..

[39] Cai Y., Sun M., Corke H. (2003). Antioxidant activity of betalains from plants of the amaranthaceae. J. Agric. Food Chem..

[40] Sakihama Y., Kato T., Sawatdee S., Yakushi Y., Asano J., Hayashi H., Goto Y., Hashimoto M., Hashidoko Y. (2023). Isolation of High-Purity Betanin from Red Beet and Elucidation of Its Antioxidant Activity Against Peroxynitrite: An In Vitro Study. Int. J. Mol. Sci..

[41] Muramatsu D., Uchiyama H., Higashi H., Kida H., Iwai A. (2023). Effects of heat degradation of betanin in red beetroot (*Beta vulgaris* L.) on biological activity and antioxidant capacity. PLoS ONE.

[42] Gliszczyńska-Swigło A., Szymusiak H., Malinowska P. (2006). Betanin, the main pigment of red beet: Molecular origin of its exceptionally high free radical-scavenging activity. Food Addit. Contam..

[43] Tsai P.J., Sheu C.H., Wu P.H., Sun Y.F. (2010). Thermal and pH stability of betacyanin pigment of Djulis (*Chenopodium formosanum*) in Taiwan and their relation to antioxidant activity. J. Agric. Food Chem..

[44] Sadowska-Bartosz I., Bartosz G. (2021). Biological Properties and Applications of Betalains. Molecules.

[45] Vieira Teixeira da Silva D., dos Santos Baião D., de Oliveira Silva F., Alves G., Perrone D., Mere Del Aguila E., Paschoalin V.M.F. (2019). Betanin, a Natural Food Additive: Stability, Bioavailability, Antioxidant and Preservative Ability Assessments. Molecules.

[46] Airen Z., Yu Y., Jing L., Xu B., Yu X., Qiu Y., Cao S. (2011). Study on the relation of structure and antioxidant activity of isorhamnetin, quercetin, phloretin, silybin and phloretin isonicotinyl hydrazone. Free Rad. Antiox..

[47] Mhiri R., Soltani S., Baccouche N., Allouche N., Hichem B.S. (2024). Chemical composition and biological activities of essential oils from two *Opuntia* species growing in Tunisia: An in vitro and in silico studies. J. Essent. Oil Bear. Plants.

[48] Dubey K., Dubey R., Gupta R., Gupta A. (2020). α-Amylase, α-glucosidase and aldose reductase inhibitory potential of betanin for the management of diabetes and its complications. J. Adv. Sci. Res..

[49] Lee H.S., Choi S.M., Lim S.H., Choi C.-I. (2023). Betanin from Beetroot (*Beta vulgaris* L.) Regulates Lipid Metabolism and Promotes Fat Browning in 3T3-L1 Adipocytes. Pharmaceuticals.

[50] Serrano-Sandoval S.N., Parralejo-Sanz S., Lobo M.G., Cano M.P., Antunes-Ricardo M. (2025). A bio-guided search of anti-steatotic compounds in *Opuntia stricta* var. *dilleniii* by fast centrifugal partition chromatography. Food Chem..

[51] Tundis R., Loizzo M.R., Statti G.A., Menichini F. (2007). Inhibitory effects on the digestive enzyme alpha-amylase of three *Salsola* species (Chenopodiaceae) in vitro. Pharmazie.

[52] Metibemu D.S., Saliu J.A., Metibemu A.O., Oluwadahunsi O.J., Oboh G., Omotuyi I.O., Akinloye O.A. (2016). Molecular Docking Studies of Isorhamnetin from *Corchorus olitorius* with Target Alpha-Amylase Related to Type 2 Diabetes. J. Chem. Pharm. Res..

[53] Lee D., Park J.Y., Lee S., Kang K.S. (2021). In Vitro Studies to Assess the α-Glucosidase Inhibitory Activity and Insulin Secretion Effect of Isorhamnetin 3-*O*-Glucoside and Quercetin 3-*O*-Glucoside Isolated from *Salicornia herbacea*. Processes.

[54] Zhang X., Jia Y., Ma Y., Cheng G., Cai S. (2018). Phenolic Composition, Antioxidant Properties, and Inhibition toward Digestive Enzymes with Molecular Docking Analysis of Different Fractions from *Prinsepia utilis* Royle Fruits. Molecules.

[55] González-Arceo M., Gomez-Lopez I., Carr-Ugarte H., Eseberri I., González M., Cano M.P., Portillo M.P., Gómez-Zorita S. (2023). Anti-Obesity Effects of Isorhamnetin and Isorhamnetin Conjugates. Int. J. Mol. Sci..

[56] Türkyilmaz M., Tagı S., Dereli U., Ozkan M. (2013). Effects of various pressing programs and yields on the antioxidant activity, antimicrobial activity, phenolic content and colour of pomegranate juices. Food Chem..

[57] Loizzo M.R., Leporini M., Sicari V., Falco T., Pellicanò M.T., Tundis R. (2017). Investigating the in vitro hypoglycaemic and antioxidant properties of *Citrus* × *clementina* Hort. Juice. Eur. Food Res. Technol..

[58] Khatabi O., Hanine H., Elothmani D., Hasib A. (2016). Extraction and determination of polyphenols and betalain pigments in the Moroccan Prickly pear fruits (*Opuntia ficus indica*). Arab. J. Chem..

[59] Loizzo M.R., Tundis R., Leporini M., D’Urso G., Gagliano Candela R., Falco T., Piacente S., Bruno M., Sottile F. (2021). Almond (*Prunus dulcis* cv. Casteltermini) Skin Confectionery By-Products: New Opportunity for the Development of a Functional Blackberry (*Rubus ulmifolius* Schott) Jam. Antioxidants.

