# Supplementary material for: Opuntia stricta (Haw.) Fruit Pulp and Seeds as Source of Bioactive Phytochemicals with Promising Functional Properties
Source: Molecules. 2025 Apr 1;30(7):1580. doi: 10.3390/molecules30071580 (PMC11990217; doi:10.3390/molecules30071580)
Supplement: Supplementary file 1 [file molecules-30-01580-s001.zip › molecules-3555928-supplementary.pdf]

Supplementary Materials

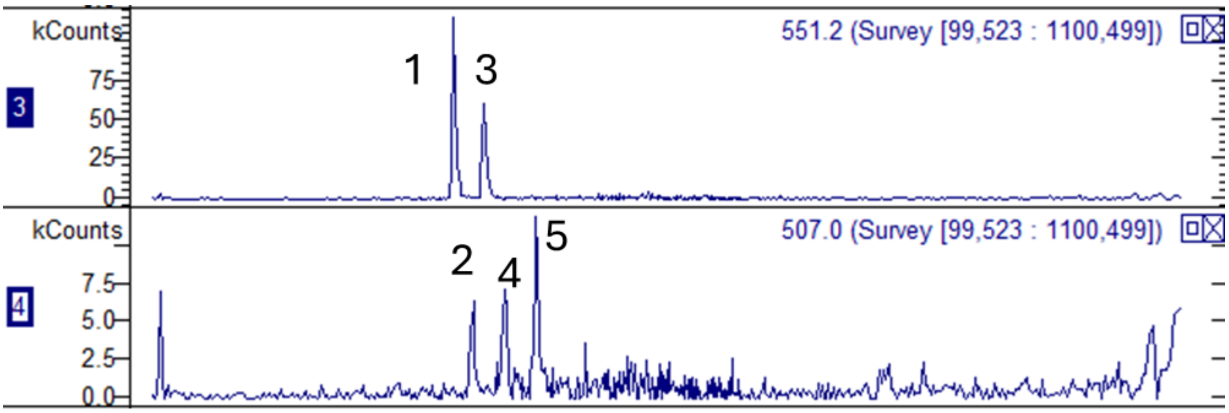

Figure S1. LC-MS chromatograms of the ions $[M+H]^+$ 551 and 507 of the OSF extract.

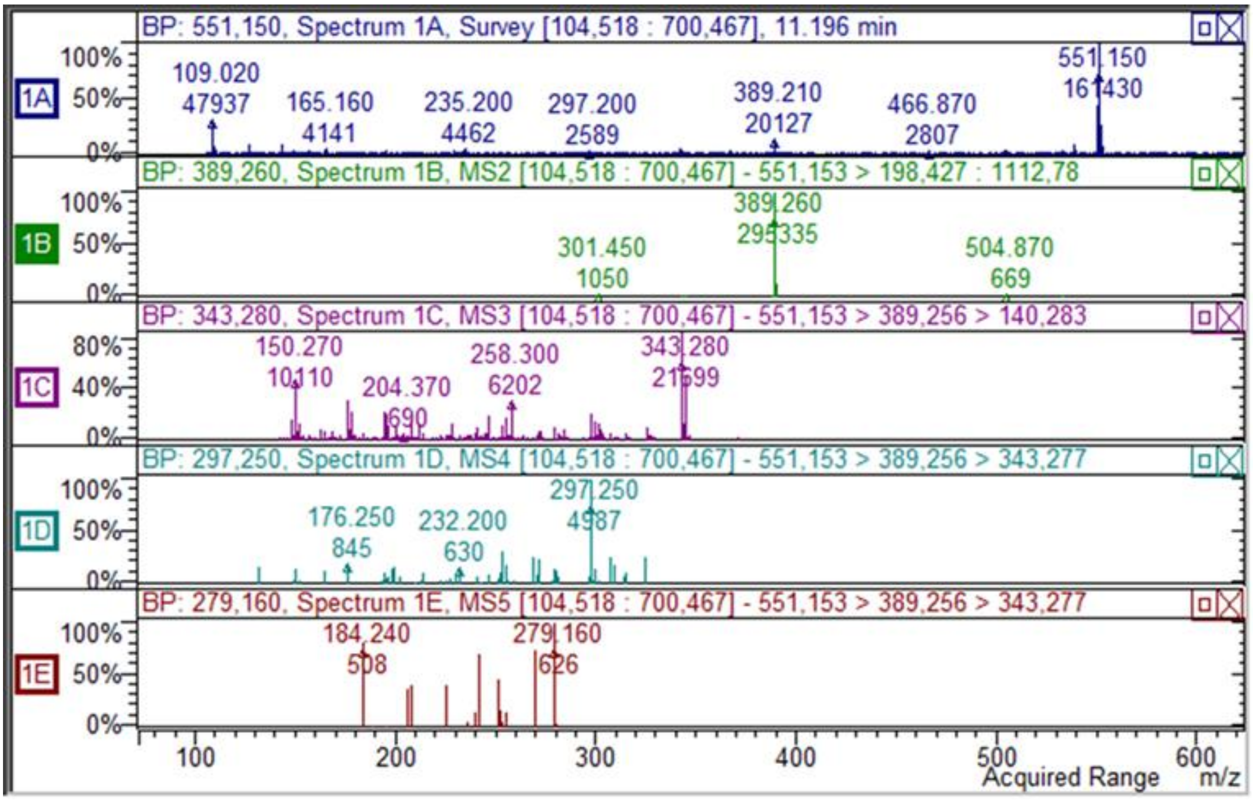

Figure S2. Mass spectra of ion 551 *m*/*z*.

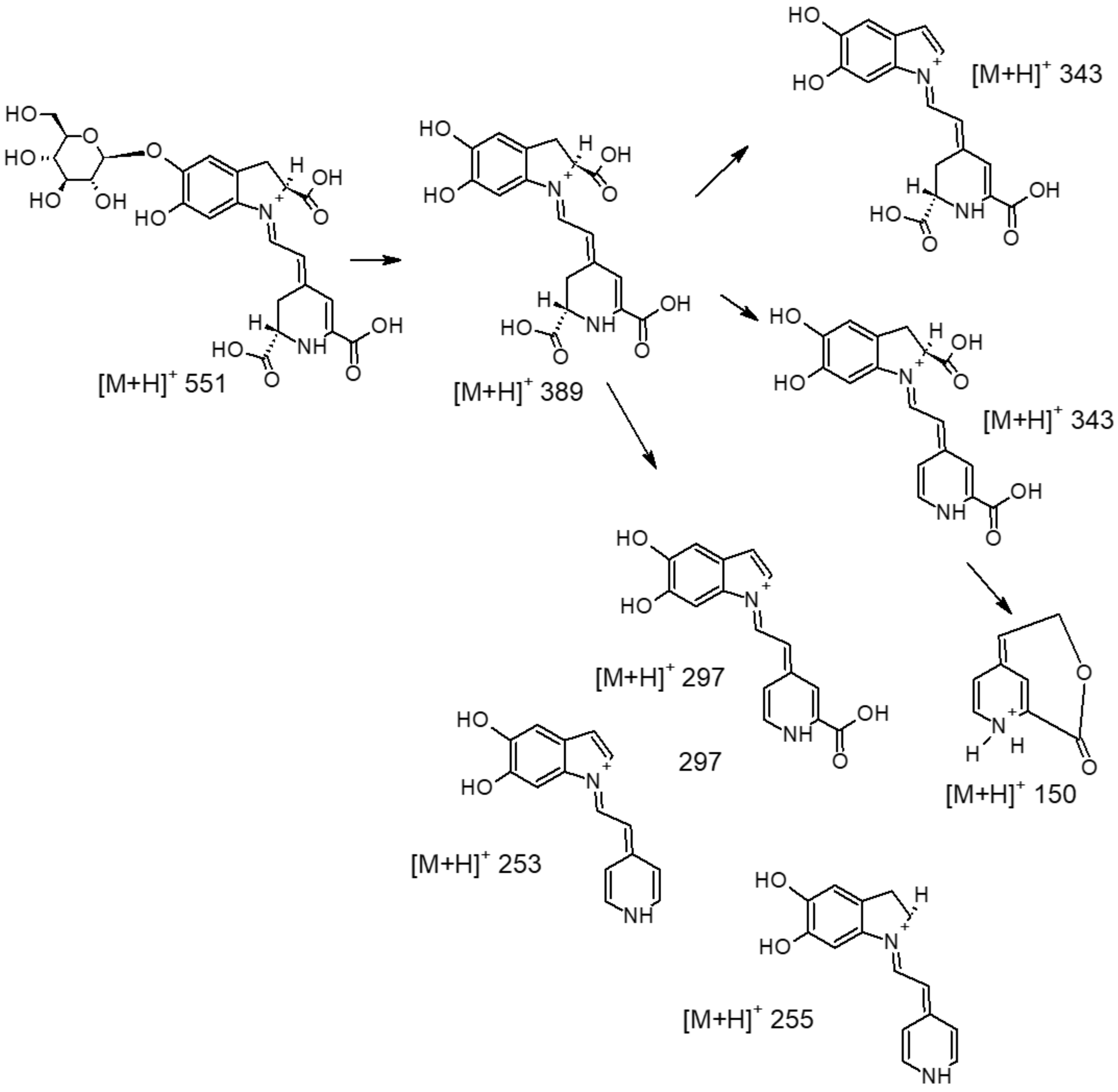

Figure S3. Proposed mass fragmentation pathway of the ion at *m*/*z* 551.
